# Metamaterial Inspired Varactor-Tuned Antenna with Frequency Reconfigurability and Pattern Diversity

**DOI:** 10.3390/s24061956

**Published:** 2024-03-19

**Authors:** Jiahao Zhang, Buyun Wang, Sen Yan, Wei Li, Guy A. E. Vandenbosch

**Affiliations:** 1 National Key Laboratory of Electromagnetic Energy, Naval University of Engineering, Wuhan 430030, China; jiahao.z@hotmail.com (J.Z.); rf_liwei@163.com (W.L.); 2 Faculty of Electronics and Information, Xi’an Jiaotong University, Xi’an 710049, China; wby3135134162@stu.xjtu.edu.cn; 3 ESAT-WaveCore Research Division, Department of Electrical Engineering, Katholieke Universiteit Leuven, 3001 Leuven, Belgium; guy.vandenbosch@esat.kuleuven.be

**Keywords:** metamaterials, multi-functional components, smart antennas, reconfigurable antennas

## Abstract

A metamaterial-inspired varactor-tuned antenna with frequency reconfigurability and pattern diversity is designed. Two different versions of a reconfigurable structure are integrated into a single antenna to excite two different orthogonal patterns, which realizes pattern diversity for MIMO applications. The outer annular Composite Right-/Left-Handed Transmission Line (CRLH-TL) works at the 1 mode and provides a broadside pattern, and the inner circular radiator loaded with split ring resonators (SRR) operates at the 0 mode and radiates an omnidirectional pattern, which realizes pattern diversity. By using surface-mounted varactors, the operating frequencies for the two radiation patterns can be tuned over a wide frequency range, from 1.7 GHz to 2.2 GHz, covering the 1.71–2.17 GHz LTE band, and a low mutual coupling between the two radiators is achieved. The antenna has also been prototyped. The measured results are in good agreement with the simulation results, verifying the proposed concept. The dual-mode MIMO system equipped with the proposed antenna elements is discussed within the context of a 3-D channel model, and it shows a superior array compactness and spectral efficiency (SE) performance compared to scenarios with single-mode elements.

## 1. Introduction

With the rapid development of modern wireless technologies, complex wireless networks that integrate multiple devices and functions have come into the picture. Such systems mainly require circuits and components that have the capability of dealing with different standards. As a crucial part of the wireless network, antennas act as edge gateways to connect various applications. To meet the booming connection requirements, antennas integrated with multiple functions become one of the key enabling technologies [1].

**Figure 1:**
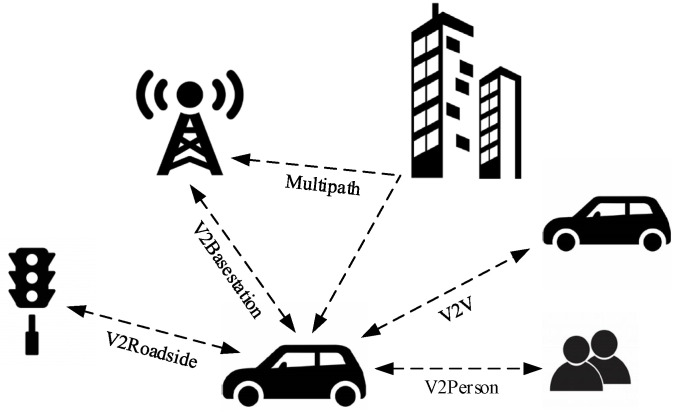
Application scenario for complex wireless networks.

An application scenario of an Internet of Things (IoT) wireless network is shown in Figure 1. A vehicle or a passenger can communicate with adjacent vehicles, passengers, roadside infrastructure, as well as the base station. Various connecting standards coexist in this scenario, and the connecting channel is complex with multipath effects. On the one hand, the system performance is limited by degradation factors like scattering, reflection, refraction, and diffraction. Antennas that can combat the effects of channel fading are thus in high demand [2]. On the other hand, with multiple communication network deployments, antennas with multifunctional properties are desirable for more wireless applications [3].

As a key benefit of Multiple-Input-Multiple-Output (MIMO) systems, antenna diversity can be realized by (1) frequency diversity, (2) spatial diversity, (3) temporal diversity, (4) polarization diversity, and (5) pattern diversity [4,5,6,7]. Pattern diversity has the ability to increase the system capacity and the link reliability by reducing the MIMO channel correlation. The patch-like radiation pattern (broadside) and the dipole-like radiation pattern (omnidirectional) are commonly combined when implementing pattern diversity [8,9] since they have “orthogonal” polarizations and main beam directions. However, the reported pattern diversity antennas mainly operate at fixed frequencies, which restricts their applications.

As the bandwidth demand for high-performance communication systems increases, the use of reconfigurable antennas for future wireless systems is emerging. A system equipped with frequency-tunable components can be implemented to increase the working band to cover as many commercial bands as possible [10]. It can also be combined with cognitive radio [11] to achieve dynamic spectrum allocation [12]. Furthermore, it has potential in frequency-diverse systems, where the different elements carry signals at different frequencies [13].

It is quite straightforward to design antennas that integrate pattern diversity and frequency tuning ability. Efforts have been made to study frequency and pattern reconfigurable antennas [14]. However, these antennas only reconfigure their operation frequencies in discrete bands. Continuous frequency tuning is not achieved. Some work proposed frequency and pattern reconfigurable antennas with continuous frequency tuning capability [15,16,17,18,19,20]. Liquid crystal technology was used to tune resonances [16]. However, the frequency tuning range is quite small. A multi-reconfigurable CLL-loaded planar monopole was proposed without pattern diversity [17]. 60 PIN-diode switches were used, which makes the antenna very complicated [18]. They achieved beam-steered pattern reconfiguration at the cost of a quite complex array or periodic structure. This makes them unsuitable as elements in a MIMO array. A frequency-reconfigurable antenna with two different patterns was proposed [21], but the two radiation patterns operate in different frequency bands. An antenna can switch its pattern between omnidirectional and end-fire patterns [20], but again, the frequency tuning range is very small. Pattern diversity was only achieved by combining two or more pattern reconfigurable antennas [16,22], which means these designs cannot be used as a MIMO antenna element to achieve pattern diversity. As far as the authors know, single antenna packaging frequency reconfigurability and dual-mode pattern diversity have not been reported yet in the literature.

Metamaterial concepts provide a potential way to realize the combination of frequency reconfigurability and pattern diversity. With the development of metamaterials, novel approaches to antenna design are introduced [23,24,25,26,27,28,29,30,31]. As a new research direction, reconfigurable metamaterials have spawned considerable research interests. They have been implemented in numerous applications [24,25]. An additional set of degrees of freedom is obtained by using reconfigurable metamaterials because of their unique and novel electromagnetic field properties in our previous work [26]. Ref. [27] proposed a metamaterial absorber, not an antenna. Refs. [28,31] proposed an antenna with metamaterials to realize high gain, high isolation, and wideband, not a reconfigurable antenna. Ref. [29] proposed a sensing system with tunable ultra-sharp Fano resonance peaks, not a tunable antenna. Ref. [30] proposed a metamaterial antenna with high isolation, not a reconfigurable antenna.

In this paper, an integrated antenna component, which contains frequency reconfigurability and pattern diversity, is realized based on a varactor-tuned metamaterial structure. Two different versions of a metamaterial-based reconfigurable structure are integrated into a single antenna to excite two different radiating modes: the 1 mode and the 0 modes, respectively. The outer radiator is an annular (composite-right/left-handed transmission-line) CRLH-TL radiator, which works at the 1 mode and provides a broadside radiation pattern based on the analysis of the dispersion curves and the equivalent circuits model. The left-handed capacitance is changed by using the varactor diodes to tune the dispersion curves of the annular CRLH-TL. The frequency tunability of the 1 mode is thus achieved. The inner radiator is a circular patch radiator loaded with split ring resonators (SRRs), which works at the 0 mode and provides an omnidirectional radiation pattern. The miniaturized inner circular radiator is arranged inside the outer annular radiator. Varactor diodes are loaded to change their resonances. In this way, the frequency tuning properties are achieved simultaneously for both radiators with different radiation patterns. The antenna is able to change its operating frequency over a wide tuning range, from 1.7 GHz to 2.2 GHz (25.6%), covering the 1.71–2.17 GHz LTE band, and the radiations for two ports are kept with tuning the operating frequency. The mutual coupling between the two ports is quite low. Despite the complex reconfigurability of the designed antenna, a simple DC bias network is realized by “borrowing” the antenna radiation structure and utilizing the symmetrical arrangement of the varactors. To the best of the authors’ knowledge, this is the first time that a reconfigurable annular CRLH-TL metamaterial has been proposed in an antenna. A 32-element MIMO array equipped with the proposed antenna element is analyzed in the context of a 3-Dimensional (3D) channel model, which includes the antenna element’s radiation pattern. Compared to a single-mode 32-element array, a better spectral efficiency (SE) is obtained.

## 2. Operating Mechanism of Dual Mode Antenna

### 2.1. CRLH-TL Based Annular Radiator with Broadside Radiation Pattern

**Figure 2:**
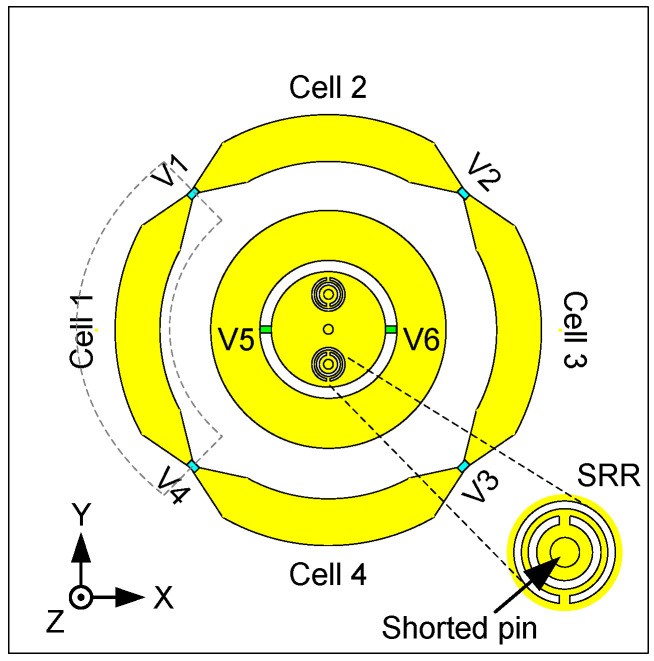
Outer radiator: CRLH-TL based annular radiator. Inner radiator: miniaturized SRR loaded circular radiator. The yellow indicates the radiator is located on a grounded substrate. V1–V6 indicate the varactors.

**Figure 3:**
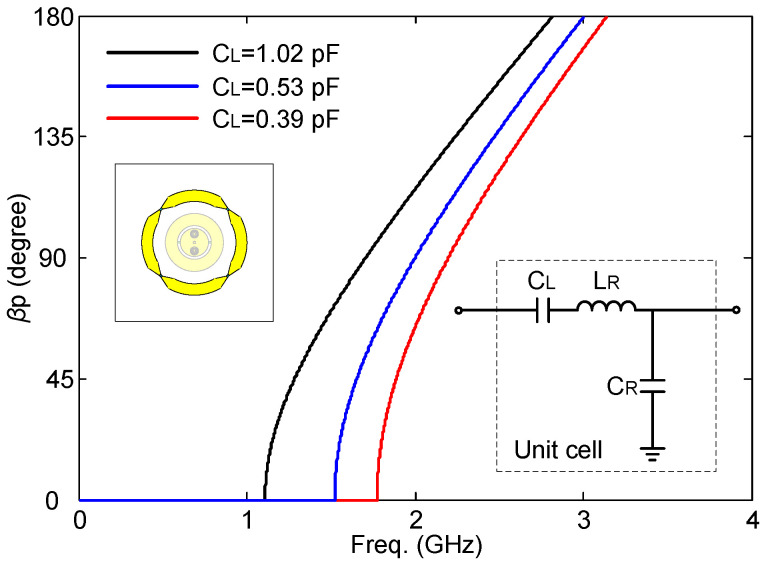
Dispersion curve of the equivalent circuit. Insert at the bottom right corner is the equivalent circuit model of the unit cell.

CRLH-TL, a type of planar metamaterials, has been widely used in circuit design and antenna design [32,33] since it can be easily realized, for example, by Printed Circuit Board (PCB) technologies. The CRLH-TL-based antennas can be not only in positive mode but also in negative mode and zeroth mode. By tuning the dispersion properties, the resonant frequencies of a CRLH-TL-based radiator can be changed. As a result, by using the CRLH-TL structures, more flexibility and freedom in the antenna design are obtained than with other traditional methods. CRLH-TLs have been studied in antenna design to excite a patch-like broadside radiation pattern for both the rectangular topology [34] and the annular topology [35]. Most of the designs work at a fixed resonant frequency. A novel frequency reconfigurable antenna is proposed based on an annular shape combined with CRLH-TL. A simple, via-free topology of the annular CRLH-TL is developed for robustness and easy fabrication. The proposed topology is shown in Figure 2 (outer annular radiator), and the equivalent circuit model of the CRLH-TL unit cells is shown in Figure 3. The annular TL structure provides the shunt capacitance (*C_R_*, right-handed capacitance) and the series inductance (*L_R_*, right-handed inductance). The gap between the annular transmission lines provides the series capacitance (*C_L_*, left-handed capacitance). In this topology, no left-handed inductance (*L_L_*, left-handed inductance) occurs. This is an unbalanced CRLH-TL that only has one left-handed parameter (*C_L_*). The dispersion curve of this CRLH-TL is presented [23]:

(1) $$\beta p=s\left(\omega \right)\sqrt{\omega^{2}L_{R}C_{R}+\frac{1}{\omega^{2}L_{L}C_{L}}-\left(\frac{L_{R}}{L_{L}}+\frac{C_{R}}{C_{L}}\right)}\;$$

where

(2) $$s\left(\omega \right)=\left\{\begin{array}{c} -1\;if\;\omega <\omega_{\tau 1}=\min\left(\frac{1}{\sqrt{L_{R}C_{L}}},\frac{1}{\sqrt{L_{L}C_{R}}}\right)\\ \;1\;if\;\omega >\omega_{\tau 2}=\max\left(\frac{1}{\sqrt{L_{R}C_{L}}},\frac{1}{\sqrt{L_{L}C_{R}}}\right) \end{array}\;\right.$$

and *p* is the physical length of the annular unit cell.

The resonant frequencies of the annular CRLH-TL are expressed as

(3) Nβp=2nπ

where *N*, β, and *n* are the number of unit cells, the propagation phase constant of the CRLH-TL, and the resonant mode number, respectively. In the proposed design, *N* equals 4. In order to achieve a broadside radiation pattern, the first resonant mode (*n* = 1) was used.

From the discussion above, it is seen that by reconfiguring the dispersion properties, the resonant frequencies of the CRLH-TL can be tuned very easily. In our model, three main parameters can be tuned to control the dispersion properties: *C_R_*, *L_R_*, and *C_L_*. In the proposed design, the left-handed capacitance *C_L_* is chosen to be tuned, by loading a varactor diode over the slot between two annular unit cells.

The objective frequency tuning range of this design is from 1.7 GHz to 2.2 GHz. The circuit model (Figure 3) is chosen to fit these resonant frequencies. To achieve this goal, Formulas (1)–(3) are used. The obtained values of the components are given in Table 1. The dispersion properties of the annular structure are illustrated in Figure 3. It is clearly shown that the resonant frequency (1 mode) can be tuned by changing the *C_L_*. When the *C_L_* = 1.02 pF, 0.53 pF, and 0.39 pF, the first mode occurs at 1.7 GHz, 2.0 GHz, and 2.2 GHz, respectively. The frequency reconfiguration with a broadside radiation pattern was obtained like this.

### 2.2. Miniaturized SRR Loaded Circular Radiator with Omnidirectional Radiation Pattern

The circular patch antenna with an omnidirectional radiation pattern was studied in [36]. When a circular patch radiator is fed by a coaxial probe from its geometric center, the TM_0m0_ modes can be excited. The omnidirectional pattern is achieved due to the circular symmetry in the physical topology. The inner radiator should be smaller than the outer annular radiator, while they have to operate at the same frequency band. Therefore, one of the big problems is to design an inner circular radiator with a compact size while keeping the resonances for both the circular and the annular radiators the same. For this reason, an SRR was loaded to miniaturize the inner circular radiator. Then, the frequency reconfigurability was achieved by introducing varactor diodes to the inner circular radiator.

**Figure 4:**
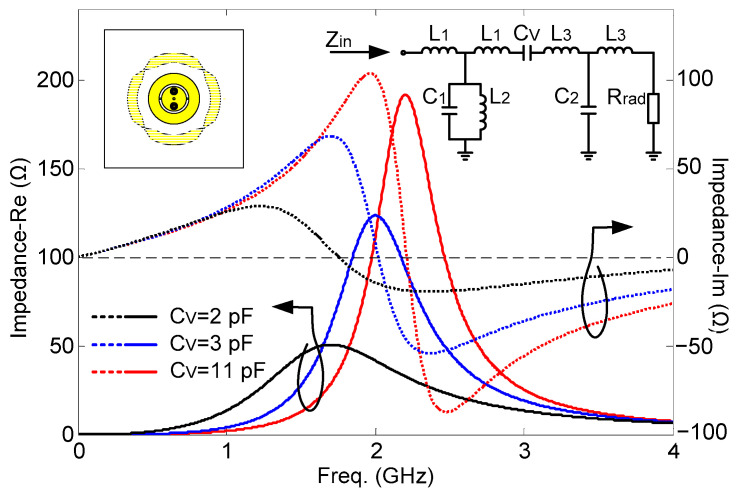
Input impedance characteristics: real part and imaginary part. Insert at the top right corner is the equivalent circuit model of the circular radiator.

The topology is illustrated in Figure 2 (inner circular radiator). In order to reveal the operating mechanism and the physical meaning behind the proposed topology, the equivalent circuit model of the inner circular patch radiator is depicted in Figure 4. The circular patch provides the series inductances *L*_1_ and *L*_3_ and the shunt capacitances *C*_1_ and *C*_2_. The symmetrically arranged shorted pins provide the shunt inductance *L*_2_. The SRRs around the shorted pins increase the inductance *L*_2_. The annular gap is bridged by two varactors symmetrically positioned left and right. Both the gap and the varactors provide the series capacitance *C_V_*. The radiation resistance *R_rad_* can be estimated by regarding the outer or the inner radiator as an annular ring microstrip antenna with a large radius or a small radius, respectively. The values of the equivalent lumped elements are chosen to fit the needed resonances of the proposed antenna, shown in Table 1. As we can see from the impedance characteristic in Figure 4, the resonance can be tuned by changing *C_V_*. When *C_V_* = 2 pF, 3 pF, and 11 pF, the resonances occur at 1.7 GHz, 2.0 GHz, and 2.2 GHz, respectively.

## 3. Fabrication and Measurement

### 3.1. Integration of the Proposed Dual-Mode Antenna

The annular radiator and the circular radiator described in the previous section are integrated into a single antenna component. A frequency-tuning antenna with two ports and two orthogonal patterns is thus achieved. The inner circular radiator is arranged inside the outer annular radiator. In order to excite the 1 mode, an aperture-coupled feeding topology is used to feed port 2 (outer radiator). Port 1 (inner radiator) is fed by a probe.

**Figure 5:**
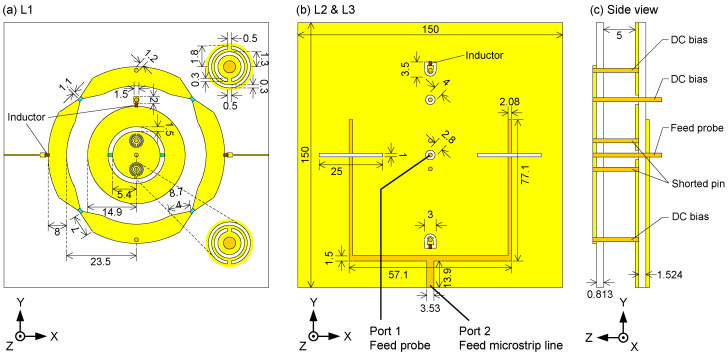
Topology of the proposed antenna: (**a**) radiator layer (*L*1), (**b**) ground layer and feeding network layer (*L*2 & *L*3), (**c**) side view.

The detailed design is given in Figure 5. The designed antenna contains two substrate layers of the type RO4003 (*ε_r_* = 3.5, *tanδ* = 0.0027). The thickness of the upper substrate and the lower substrate are 0.813 mm and 1.524 mm, respectively. The annular radiator structure and the circular radiator structure are printed on the upper substrate (*L*_1_). The top layer and the bottom layer of the lower substrate are defined as L_2_ and L_3_, respectively. *L*_2_ locates the antenna ground with two coupling apertures, and L_3_ locates the feeding structures of port 2. The upper substrate and the lower substrate are spaced by a 5 mm air gap, which can enhance the bandwidth of the proposed antenna.

The initial dimensions of the antenna were obtained by using the circuit models explained before in combination with empirical equations [37]. Our previous experience proved to be very beneficial here. The circuit models were thus used to have a first rough idea, not to predict the sophisticated antenna performance. The initial values of all the parameters are determined by the dispersion curves of the equivalent circuits for the outer and inner radiators, as shown in Figure 3 and Figure 4. Then, the topology of the antenna is modeled based on the dispersion curves and the impedance matching for feeding networks. At last, the optimization is applied in CST Microwave Studio, and the genetic algorithm is used in this work. The maximum number of iterations is 30, and the maximum number of solver evaluations is 497.

### 3.2. Varactors and DC Bias

Four varactor diodes (*V*1–*V*4) are loaded on the slots between the annular unit cells of the outer radiator. As discussed in the previous section, in order to make the outer radiator work at the targeted frequency, a very small capacitance is needed. Therefore, MACOM MGV125-21 varactor (M/A-COM Technology Solutions Inc products, Lowell, MA, USA) diodes are used [38]. The four varactors are separated into two sets and placed inversely, i.e., the polarity of *V*1&*V*2 and *V*3&*V*4 are opposite. The cathodes of *V*1 and *V*2 are connected to Cell 2 and then connected to the ground via an RF choke inductor (inductance 51 nH, series resistance 1 Ohm). The cathodes of *V3* and *V4* are connected to Cell 4 and then connected to the ground via the same type of RF choke inductance as mentioned above. The anodes of *V*1 and *V*2 are connected to two different cells: Cell 1 and Cell 3, respectively. Similarly, the anodes of *V*3 and *V*4 are connected to Cell 3 and Cell 1, respectively. They are all biased via the same type of RF choke inductor. The DC bias network is thus simplified by this arrangement since only two bias networks are used to bias the four varactors.

**Figure 6:**
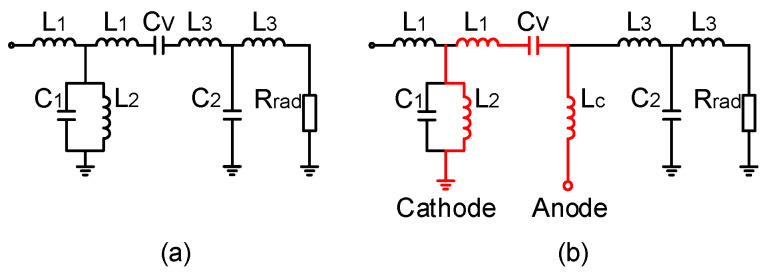
(**a**) Circuit model of the circular radiator, (**b**) circuit mode with the DC bias network. The red lines indicate the DC network. LC indicates RF choke inductor.

Two varactor diodes (Infineon BB857 silicon tuning diode [39], produced by Infineon, Munich, Germany) are symmetrically arranged over the slot of the circular radiator. The ground of the DC bias network and the ground of the antenna radiator are connected and form the ground connection for the diode cathodes via the shorted pins. The anodes of the varactor diodes are biased via the above-mentioned type of RF choke inductor. Note that the shorted pins are not only used to provide the shunt inductance L2 but also utilized to bias the inner two varactors, see Figure 6. This way of working simplifies the bias topology.

**Figure 7:**
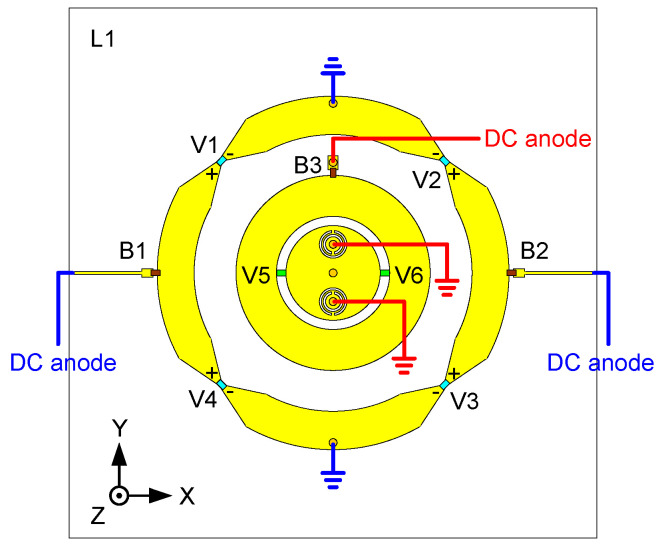
DC bias setup. *B1*–*B3* indicate the RF choke inductors.

The bias network for all the varactors is depicted in Figure 7. In summary, the bias network is not very complex and has very little impact on the antenna performance despite the complicated controlling requirements for six varactors.

## 4. Prototype Performance

**Figure 8:**
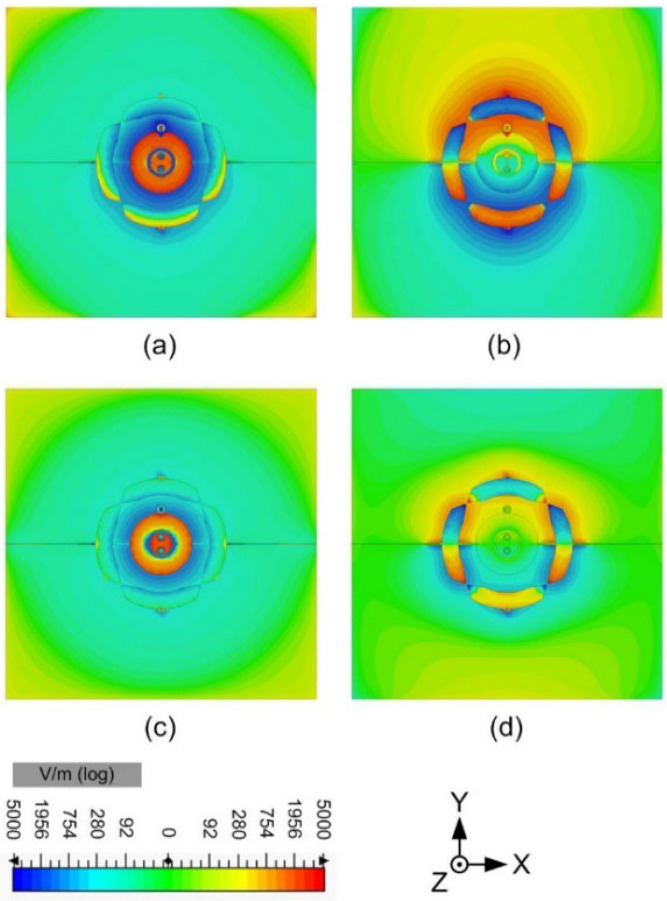
Electric field distribution (Z component of the E-field, 1 mm above the radiator). (**a**) port 1 at 1.7 GHz, (**b**) port 2 at 1.7 GHz, (**c**) port 1 at 2.2 GHz, (**d**) port 2 at 2.2 GHz.

**Figure 9:**
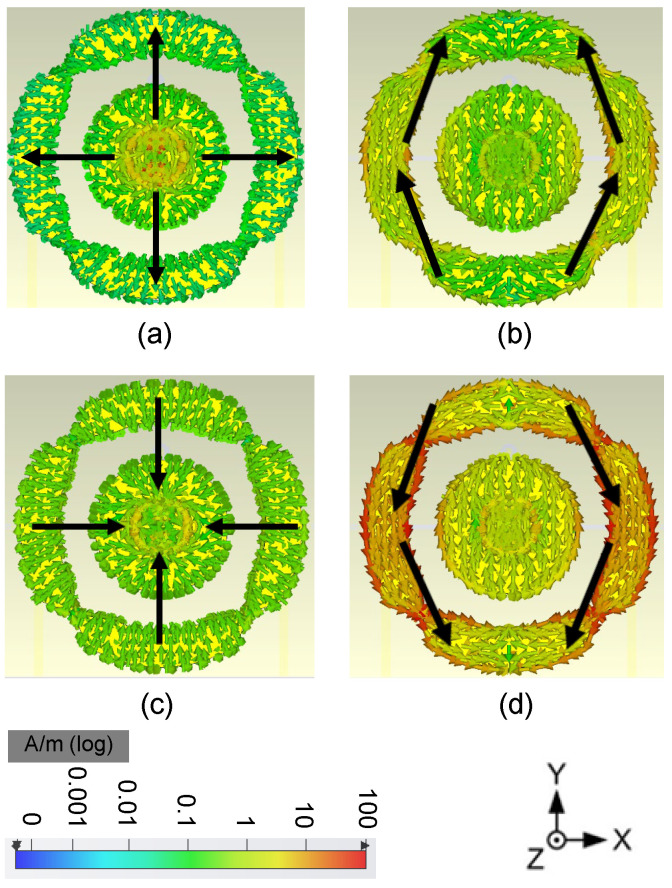
Currents distribution. (**a**) port 1 at 1.7 GHz, (**b**) port 2 at 1.7 GHz, (**c**) port 1 at 2.2 GHz, (**d**) port 2 at 2.2 GHz.

**Figure 10:**
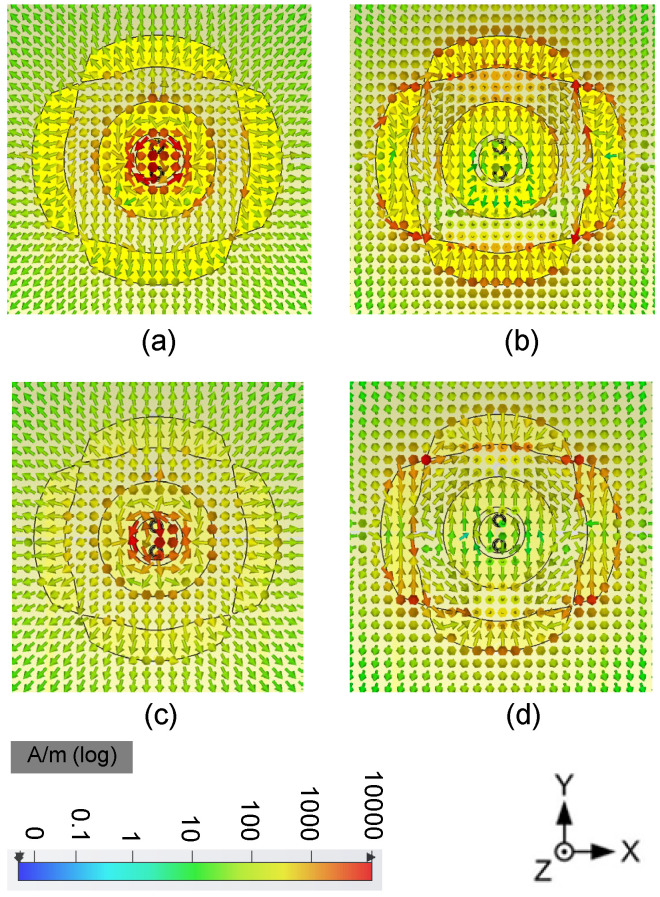
Power flow. (**a**) port 1 at 1.7 GHz, (**b**) port 2 at 1.7 GHz, (**c**) port 1 at 2.2 GHz, (**d**) port 2 at 2.2 GHz.

The simulated electric field (E-field) distribution of the proposed antenna is shown in Figure 8, and the current distribution is shown in Figure 9. The proposed antenna is to be designed as a pattern diversity antenna, which will be applied on base stations, so the E-field and current distributions are given at port 1 and port 2, respectively. It is easily observed that the E-field distribution matches the 1 mode and 0 mode for the annular radiator and the circular radiator, respectively. The power flow is given in Figure 10 to show the mode and Poynting vector. It can be seen that it is in keeping with the E fields in Figure 8 and currents in Figure 9.

**Figure 11:**
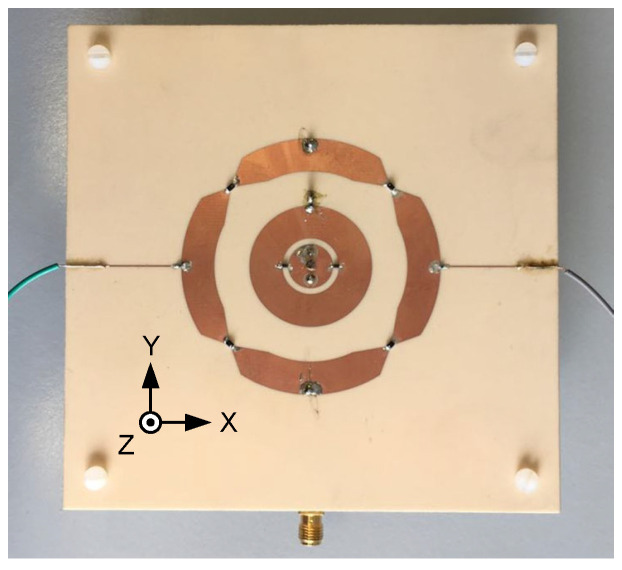
The fabricated antenna.

To validate the proposed concept, a prototype of the designed antenna element was fabricated and assembled, see Figure 11. The reflection coefficient and the mutual coupling were measured with a Vector Network Analyzer. The radiation performances of the antenna were tested in the anechoic chamber for each feed port, while the other feed port was matched with a 50 Ω load.

**Figure 12:**
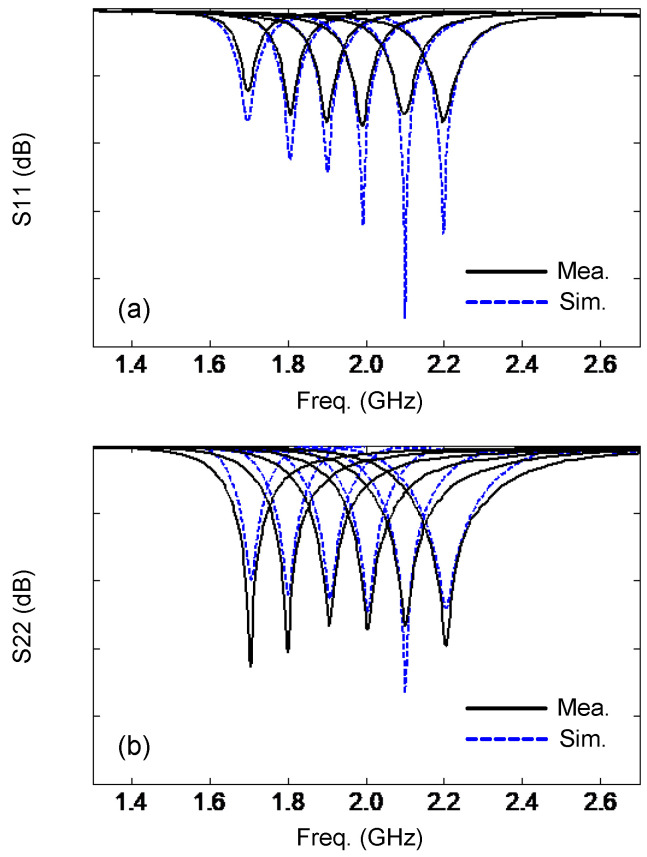
Reflection coefficients: (**a**) port 1, (**b**) port 2. The six resonances are at 1.7, 1.8, 1.9, 2.0, 2.1, and 2.2 GHz, respectively.

The measured and simulated reflection coefficients are given in Figure 12. The measurements show that the resonant frequency tuning range for both ports is very wide: from 1.7 GHz to 2.2 GH, covering the 1.71–2.17 GHz LTE band (e.g., the 1.7 GHz AWS-1 band, the 1.8 GHz DCS band, the 1.9 GHz PCS band, and the 2.1 GHz IMT band). The bandwidth (10 dB return loss criterion) for both ports is larger than 25 MHz in all cases. This is wide enough for the channel bandwidth requirements of LTE. Table 2 summarizes the values of the varactor capacitance versus frequency. Note that the application of the proposed antenna is not limited to LTE. It can be rescaled to cover other needed bands [40]. Although it is not easy to extend the design to higher frequencies when normal varactors are used, by using micro-electro-mechanical-systems (MEMS), a varactor with very small capacitance can be realized [41], needed at 6 GHz and higher, as considered in 5G systems.

**Figure 13:**
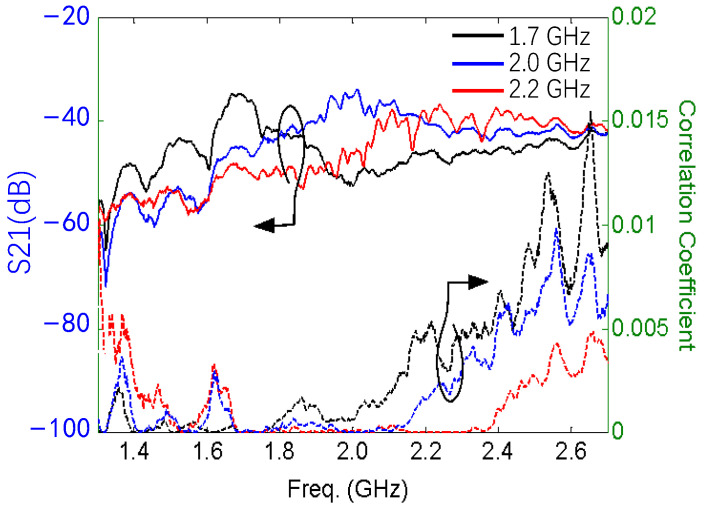
Mutual coupling and correlation coefficient. The solid line indicates the mutual coupling, and the dashed line indicates the correlation coefficient. The black line represents 1.7 GHz, the blue line represents 2.0 GHz, and the red line represents 2.2 GHz.

The mutual coupling between port 1 and port 2 is quite low, lower than −30 dB for all cases. A simple and useful way to evaluate the antenna diversity performance is to use the correlation coefficient estimated from the S-parameters [42]. Figure 13 shows the measured mutual coupling and the correlation coefficient calculated from the measured S-parameters at the tuned operational frequencies 1.7 GHz, 2.0 GHz, and 2.2 GHz. For antenna diversity, the practically acceptable correlation coefficient should be less than 0.5 [43]. In our design, the value is less than 0.02 in the entire operating band. A very low mutual coupling between the two ports and an extremely low correlation coefficient guarantee a very good diversity performance for a MIMO system.

**Figure 14:**
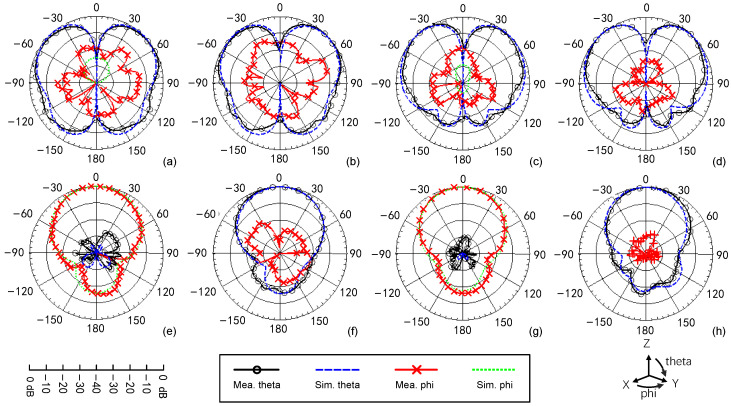
The normalized radiation patterns of the proposed antenna. Port 1 at 1.7 GHz (**a**) XZ plane, (**b**) YZ plane. Port 1 at 2.2 GHz (**c**) XZ plane, (**d**) YZ plane. Port 2 at 1.7 GHz (**e**) XZ plane, (**f**) YZ plane. Port 2 at 2.2 GHz (**g**) XZ plane, (**h**) YZ plane.

**Figure 15:**
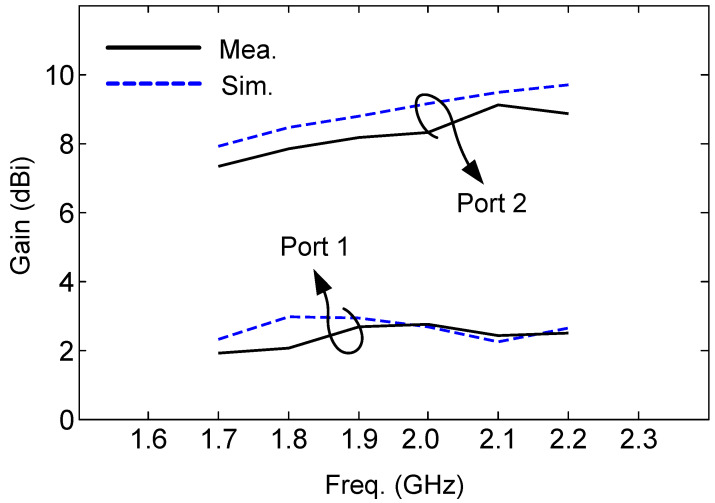
Realized gain of the antenna.

The normalized radiation patterns of both port 1 and port 2 at 1.7 GHz and 2.2 GHz are shown in Figure 14. A reasonable agreement between the measurements and simulations is obtained. It can clearly be seen that the radiation pattern is a typical omnidirectional pattern for port 1 and a typical broadside pattern for port 2. A higher gain for port 2 is observed than port 2 since the radiation pattern for port 2 is broadside, while the radiation pattern for port 1 is omnidirectional. The mode of P2 is like TM_11_ mode in an annular microstrip antenna. The annular microstrip antenna that operates on TM_11_ mode with the same size as the proposed antenna can realize a 9 dB gain at the same frequency, which is similar to the proposed antenna. As for the compatibility of impedance bandwidth and gain bandwidth, the impedance bandwidth for both ports is 25 MHz, and their gain bandwidth is higher than the impedance bandwidth. There is only Figure 12b performing a high measured X-polarization (−10 dB), and others are below −15 dB. The main reason is the influence caused by the feeding lines in measurement because the simulated X-polarization results in Figure 12 are too low. The other reason is the low gain of the omnidirectional radiations by P1, causing a slightly higher X-polarization in measurement. It is not a huge issue because the demand for X polarization is not high for indoor communications. The solutions are the following: (1) the choke ring can be set around the feeding line to weaken the influence of the feeding line; (2) shorten the length of the feeding line; (3) or design a feeding network for P1 to change the direction of the feeding line from vertical to horizontal. This point has been added in Section 4. For all broadside patterns, the front-to-back ratio (FBR) is above 15 dB. Based on the simulated 3D radiation patterns, the ECC is calculated, and they are also lower than 0.02. The realized gain of the presented design is shown in Figure 15. A little discrepancy is observed between the measured and simulated results, which is probably because of the fabrication tolerances of the antenna and the measurement errors. When the operating frequency changes to 1.7 GHz, the measured radiation efficiency for port 1 is 61.3%. When it changes to 2.2 GHz, the measured radiation efficiency for port 1 is 72.6%, and 81.2% and 90.8% at 1.7 GHz and 2.2 GHz for port 2, respectively. The simulation results are 65.9% and 81.8% at 1.7 GHz and 2.2 GHz for port 1, respectively, and 82.9% and 97.3% at 1.7 GHz and 2.2 GHz for port 2, respectively. The power losses mainly occur at the varactors for both port 1 and port 2, according to simulations, but the losses are not a great deterioration (4.7% and 9.2% at 1.7 GHz and 2.2 GHz for port 1, 1.7% and 6.5% at 1.7 GHz and 2.2 GHz for port 2). Therefore, the effect of the deterioration caused by the varactors is acceptable for the proposed applications.

A brief comparison of reconfigurable antennas with pattern diversity or reconfigurability found in literature is given in Table 3. The proposed antenna realizes frequency reconfigurability with the widest relative tuning range (25.6%). In addition, the proposed antenna also realizes pattern diversity: the omnidirectional radiation pattern and the broadside radiation pattern, which was not realized in [15,16,17,18,19,20,22]. Six varactors are used to reconfigure the antenna.

## 5. MIMO System Analysis

### 5.1. 3-D Channel Model

In the literature, many channel models for MIMO systems describe the channel under the assumption of the same radiation pattern for all antenna elements. In most cases, this radiation pattern is assumed to be simply omnidirectional. In this work, the radiation pattern for both modes of the proposed antenna element was taken into account when analyzing the system model. Furthermore, the 3-D spatial evaluation by taking into account the antenna patterns as functions of elevation φ and azimuth θ was also included.

Consider a MIMO system that contains a base station (BS) equipped with *M* antenna elements and *N* single-antenna user terminals. Each antenna element *m* at the BS has an identical number *K* of modal radiation patterns $g_{m,1}(\theta ,\varphi )$, $g_{m,2}(\theta ,\varphi )$, …, $g_{m,K}(\theta ,\varphi )$. All *K* modes are simultaneously active and work in the same operational frequency band. The signals transmitted from the *N* users are described as a vector:

(4) $$x={\left[x_{1},x_{2},\ldots ,x_{N}\right]}^{T}$$

where $E\left\{{\left|x_{N}\right|}^{2}\right\}=1$. The received signal at the BS is described by

(5) $$y=HT^{1/2}x+w$$

where $y\in C^{M}$. $T=diag\left\{t_{1},t_{2},\ldots ,t_{N}\right\}$ with $t_{n}$ denoting the average transmit power of the *n*th user terminal. $w\sim CN(0,I_{M})$ is the independent identically distributed (i.i.d.) complex Gaussian distributed noise. The MIMO channel is described by the matrix $H={[h}_{1},h_{2},\ldots ,h_{N}]$, which represents the channel between the BS and the *n*th user terminal. According to the correlation-based stochastic channel model [44], the channel vector $h_{n}$ is decomposed into three factors: small-scale fading, large-scale fading, and antenna radiation pattern. Note that only the radiation patterns of the multi-mode antenna elements at the BS side were taken into account. Isotropic radiation was assumed at the user terminal side. The non-line-of-sight (NLoS) channel with *C* multipaths with the angle of arrivals $\left(\theta_{c},\varphi_{c}\right)$ is assumed.

(6) $$h_{n}=\frac{\sqrt{a_{n}}}{C}\sum_{c=1}^{C}h_{n,c}=\frac{\sqrt{a_{n}}}{C}\sum_{c=1}^{C}G\left(\theta_{c},\varphi_{c}\right)a\left(\theta_{c},\varphi_{c}\right)v_{n,c}\;$$

$a_{n}$ represents the large-scale fading and shadowing effect of the *n*th user. $G\left(\theta_{c},\varphi_{c}\right)$ represents the radiation pattern of the *M*-element MIMO array.

(7) $$G\left(\theta_{c},\varphi_{c}\right)=diag\left\{{\left(G_{1}\left(\theta_{c},\varphi_{c}\right)\right)}^{\frac{1}{2}},\ldots ,{G_{M}(\left(\theta_{c},\varphi_{c}\right))}^{\frac{1}{2}}\right\}$$

where $G_{m}\left(\theta_{c},\varphi_{c}\right)={\left[g_{m,1}(\theta_{c},\varphi_{c}),\ldots ,g_{m,K}(\theta_{c},\varphi_{c})\right]}^{T}$ represents that each BS antenna element has *K* different modes (radiation patterns). $a\left(\theta_{c},\varphi_{c}\right)$ denotes the steering vector, which is with *C* different angles of arrival (AoA) for the *n*th user terminal. When the square antenna array is assumed, the steering vector can be modeled as

(8) $$a\left(\theta_{c},\varphi_{c}\right)=vec\left\{\begin{array}{c} {\left[1,\;e^{j2\pi dsin\theta_{c}},\ldots ,e^{j2\pi d(M-1)sin\theta_{c}}\right]}^{T}\\ ⨂\left[1,\;e^{j2\pi dsin\varphi_{c}},\ldots ,e^{j2\pi d(M-1)sin\varphi_{c}}\right] \end{array}\right\}$$

where $vec\left\{\cdot \right\}$ denotes vectorization of the matrix. *d* is the electric length of the antenna element space with respect to the carrier wavelength. $v_{n,c}$ represents an i.i.d Gaussian vector with element-wise distribution as zero mean and unit variance.

### 5.2. Spectral Efficiency Analysis

To investigate the performance of the MIMO system equipped with the proposed frequency reconfigurable dual mode antenna element, we consider an NLoS scenario with *C* = 10, *M* = 32, *N* = 2, and *K* = 2 since two modes are present. The array distribution is linear for base stations, and the element spacing d = 0.85 *λ* > 0.5 *λ*, which can realize a low coupling between elements (<−20 dB). We fix the AoA φ=90°, and assume the AoA $\left|\theta \right|<90{}^{\circ}$. Note that the two radiation patterns of the proposed antenna element have the same phase center, so the phase delay of the steering vector for these two patterns is identical. The channel is considered ergodic. The channel vector for each cluster can be written as [45].

(9) $$h_{n,c}=\left[\begin{array}{c} \begin{array}{c} \begin{array}{c} {g_{1,1}(\theta )}^{\frac{1}{2}}\\ \begin{array}{c} \vdots \\ {g_{1,K}(\theta )}^{\frac{1}{2}} \end{array} \end{array}\\ \begin{array}{c} {g_{2,1}(\theta )}^{\frac{1}{2}}\\ \begin{array}{c} \vdots \\ {g_{2,K}(\theta )}^{\frac{1}{2}} \end{array} \end{array} \end{array}\\ \begin{array}{c} \ddots \\ \begin{array}{c} {g_{M,1}(\theta )}^{\frac{1}{2}}\\ \begin{array}{c} \vdots \\ {g_{M,K}(\theta )}^{\frac{1}{2}} \end{array} \end{array} \end{array} \end{array}\right]\times \left[\begin{array}{c} \begin{array}{c} 1\\ e^{j2\pi dsin\theta } \end{array}\\ \begin{array}{c} \vdots \\ e^{j2\pi d(M-1)sin\theta } \end{array} \end{array}\right]\;={\left[\begin{array}{c} g_{1,1}\left(\theta \right),\ldots ,g_{1,K}\left(\theta \right),\\ g_{2,1}\left(\theta \right)e^{j2\pi dsin\theta },\ldots ,g_{2,K}\left(\theta \right)e^{j2\pi dsin\theta },\ldots ,\\ g_{M,1}\left(\theta \right)e^{j2\pi d(M-1)sin\theta },\ldots ,g_{M,K}\left(\theta \right)e^{j2\pi d(M-1)sin\theta } \end{array}\right]}^{T}\;$$

**Figure 16:**
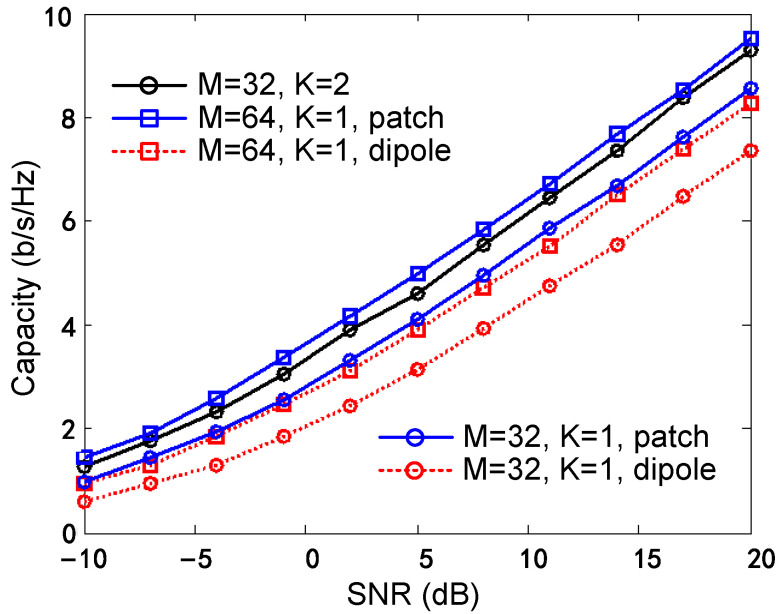
Spectral efficiency of the different scenarios.

To show the advantage of using the proposed antenna, the spectral efficiency of this system is compared with four other configurations: (1) *M* = 32, *K* = 1, patch-like mode, (2) *M* = 32, *K* = 1, dipole-like mode, (3) *M* = 64, *K* = 1, patch-like mode, (4) *M* = 64, *K* = 1, dipole-like mode. A zero-forcing (ZF) detector is used. The highest uplink spectral efficiency that can be achieved with the different scenarios is computed and shown in Figure 16. As we can see, the achievable SE of the proposed multi-mode array scenario is slightly lower than scenario (3) and higher than scenarios (1), (2), and (4). The directional antenna array shows a better SE performance than the omnidirectional antenna array. The proposed multi-mode antenna array reaches almost the same SE performance as the 64-element single-mode directional array and is thus obviously superior to the 32-element-single-mode array (both the patch-like and dipole-like array). The results indicate the advantage of using the proposed array compared to scenarios with single-mode elements in terms of the number of elements and array compactness.

## 6. Conclusions

In this paper, a smart antenna component with frequency reconfigurability and pattern diversity is proposed. Inspired by CRLH-TL concepts, a low-profile integrated antenna with two ports was obtained, which was first reported. The outer annular CRLH-TL radiator provides a broadside radiation pattern, and the inner circular patch provides an omnidirectional pattern. The frequency can be tuned by using varactors to change its impedance. The 1.7–2.2 GHz frequency tuning range is obtained, which covers the 1.71–2.17 GHz LTE band, and the radiations are kept. The mutual coupling between port 1 and port 2 is below −30 dB in the whole working band, which is an advantage of boosting the diversity performance for MIMO systems. The radiation efficiency is higher than 60% for port 1 and 80% for port 2. A 3-D channel model that takes into account the element radiation pattern has been applied. The calculated SE performance of the proposed antenna is a bit lower than twice the number of patches and higher than the same number of other single-mode antennas, which shows the advantages of using the proposed dual-mode array compared to single-mode arrays in terms of the number of elements and array compactness. The realization of frequency tunability with pattern diversity in a single component is quite promising for future smart communication or radar systems. Beyond that, the concept of continuously tuning the dispersion properties of the CRLH-TL can be stretched to other applications such as microwave circuit design, filter design, and radar design.

## Figures and Tables

**Table 1:** Parameter Values of the Equivalent Circuit.

Parameter	Value	Parameter	Value
*C_R_*	1.80 pF	*L*_1_	0.12 nH
*L_R_*	20.6 nH	*L*_2_	3.15 nH
*C*_1_	0.33 pF	*L*_3_	0.19 nH
*C*_2_	2.53 pF	*R_rad_*	38.91 Ω

**Table 2:** Values of the Varactors at Different Frequencies.

Freq. (GHz)	Port 1 (pF)		Port 2 (pF)	
	Sim.	Mea.	Sim.	Mea.
1.7	4.02	6.6	1.03	1.1
1.8	2.51	3.9	0.705	0.68
1.9	1.33	1.9	0.528	0.49
2.0	1.02	1.2	0.403	0.38
2.1	0.865	0.9	0.315	0.29
2.2	0.725	0.8	0.244	0.25

The measured values of the varactors are extracted according to capacitance-DC bias voltage curves in the specifications by using the measured DC bias voltage.

**Table 3:** Comparison of Frequency Reconfigurable Antennas with Pattern Diversity or Reconfigurability.

Ref	Antenna Type	Freq. Tuning Range		Antenna Pattern	Reconfiguration Method
		in GHz	in %		
[15]	CPW slot	2.13–2.47	14.7%	Beam steering	15 PIN diodes
[16]	Patch array	14.5–16.4	3%	Beam steering	Liquid Crystal Tech.
[17]	monopole	1.59–1.72	7.9%	Beam steering	2 switchable loops
[18]	Patch antenna	2.4–3	22.2%	Beam steering	60 PIN diodes
[19]	Slot ring array	1.68–1.83/ 5.05–6.37	8.6%/11.5%	Broadside	16 PIN diodes
[20]	Alford loop microstrip antenna	2.33-2.58	10.4%	Beam steering	3 PIN diodes
[21]	Microstrip antenna	2.7–3.3	20%	Broadside/Omnidirectional	6 Varactors
[22]	Monopole/semi ring radiator	4.27–4.94	14.5%	Omnidirectional end-fire	2 PIN diode/1 Varactors
This work	CRLH/circular antenna	1.7–2.2	25.6%	Broadside/Omnidirectional	6 Varactors

## Data Availability

Data are contained within the article.
